# The Association between Daily Dietary Intake of Riboflavin and Lung Function Impairment Related with Dibutyl Phthalate Exposure and the Possible Mechanism

**DOI:** 10.3390/nu14112282

**Published:** 2022-05-29

**Authors:** Jilei Lin, Siying Cheng, Jing Zhang, Shuhua Yuan, Lei Zhang, Jinhong Wu, Jiande Chen, Mingyu Tang, Yabin Hu, Shilu Tong, Liebin Zhao, Yong Yin

**Affiliations:** 1 Department of Respiratory Medicine, Shanghai Children’s Medical Center, School of Medicine, Shanghai Jiao Tong University, Shanghai 200127, China; jilei_lin@163.com (J.L.); zhangjing@scmc.com.cn (J.Z.); yuanshuhua@scmc.com.cn (S.Y.); zhanglei@scmc.com.cn (L.Z.); wujinhong@scmc.com.cn (J.W.); chenjiande@scmc.com.cn (J.C.); tangmingyu@scmc.com.cn (M.T.); 2 Department of Neurology, Renji Hospital, School of Medicine, Shanghai Jiao Tong University, Shanghai 200127, China; chengsiying1992@163.com; 3 Department of Clinical Epidemiology and Biostatistics, Shanghai Children’s Medical Center, School of Medicine, Shanghai Jiao Tong University, Shanghai 200127, China; huyabin@scmc.com.cn (Y.H.); tongshilu@scmc.com.cn (S.T.); 4 Shanghai Engineering Research Center of Intelligence Pediatrics (SERCIP), Shanghai 200092, China; 5 Pediatric AI Clinical Application and Research Center, Shanghai Children’s Medical Center, Shanghai 200092, China

**Keywords:** dibutyl phthalate, riboflavin, neutrophils, monocytes, National Health and Nutrition Examination Survey

## Abstract

This study aimed to evaluate the relationship between the daily dietary intake of riboflavin (DDIR) and impaired lung function associated with dibutyl phthalate (DBP) exposure. Data of 4631 adults in this national cross-sectional survey were included. Urinary mono-benzyl phthalate (MBP) was used to evaluate the level of DBP exposure. The ln-transformed urinary creatinine-corrected MBP (ln(MBP/UCr)) level was used in the statistical models. High DDIR was defined as the DDIR ≥1.8 mg per day. The results of lung function impairment and high monocytes were significantly higher in the highest MBP group compared with the lowest MBP group. A significant interaction between ln(MBP/UCr) and DDIR (*P*_interaction_ = 0.029) was detected for the risk of lung function impairment. The risk of lung function impairment (OR_quartiles4 vs. 1_ 1.85, 95% CI, 1.27–2.71; *P*_trend_ = 0.018) and high neutrophils (OR_quartiles4 vs. 1_ 1.45, 95% CI, 1.06–1.97; *P*_trend_ = 0.018) was significantly higher in the highest vs. the lowest quartile of MBP in participants with low/normal DDIR but not in in participants with high DDIR. The results of this study showed that high DDIR was associated with less lung function impairment related with DBP exposure, and the inhibiting of the neutrophil recruitment might be the potential mechanism.

## 1. Introduction

Phthalates are widely used as plasticizers in polyvinyl chloride products, medical devices, and toys [1]. The serious environmental contamination caused by phthalates have attracted extensive attention. Previous studies suggested that phthalates metabolites can be easily detected in human biospecimens due to the high exposure of phthalates in daily life [2,3]. The relationship between concentration of these metabolites and diseases was explored in many previous studies [4,5,6].

One previous study suggested that the concentration of phthalates metabolites in lung of rats is low [7], and there were limited studies on the relationship between phthalates exposure and respiratory diseases of human in past decades. In recent years, some studies have found the concentration of dibutyl phthalate (DBP) was high in indoor air, and its inhalation may contribute to more than 20% of the daily internal dose of the phthalates [8,9]. Therefore, researchers have realized that phthalates were related with the development of respiratory diseases in recent years [6,10]. Although there was no direct approach to detect phthalates concentration in human lung tissues, urinary phthalates concentration has been demonstrated to be a good biomarker of phthalates exposure. Mono-benzyl phthalate (MBP) is one of the main metabolites of DBP, and urinary MBP was usually used to evaluate the level of DBP exposure. It was reported that exposure to DBP was associated with the oxidative stress and type 2 immune response in the airway, which could contribute to the development of airway hyperresponsiveness in mice [11,12]. However, there is no population-based study to explore or verify these theories.

Riboflavin, a water-soluble vitamin, is critical for cellular growth and function. It can be obtained from various foods and participates in antioxidant and anti-inflammatory processes [13]. Previous studies showed that patients who smoke or those with COPD have lower riboflavin status compared with normal people, and COPD patients with lower riboflavin status have lower FEV1% and more serious clinical symptoms [14]. However, the role of riboflavin intake on the lung function impairment associated with phthalates exposure was unknown.

Considering the factors above, this population-based study aimed to evaluate two topics including the relationship between DBP exposure and lung function, and the role of daily dietary intake of riboflavin (DDIR) on lung function impairment related with DBP exposure in U.S. population.

## 2. Materials and Methods

### 2.1. Study Population

In this study, we used publicly available data from the National Health and Nutrition Examination Survey (NHANES) conducted by the CDC. This national cross-sectional survey was designed to collect information on the health and nutrition status of the American population. The detailed information about the NHANES study can be found online at http://www.cdc.gov/nchs/nhanes.htm (accessed on 1 February 2022). We examined national cross-sectional data in three consecutive NHANES cycles (2007–2008, 2009–2010, 2011–2012) from the NHANES database. Data on riboflavin intake, alternatives of phthalates, and lung function of adults (≥18 years old) were retrieved from the database. The informed consents of this study were obtained, and the ethics was approved by the National Center for Health Statistics Research Ethics Review Board.

### 2.2. Data Extraction

The collected data included demographic information, underlying disease, daily riboflavin intake from foods and beverages (including all types of water), urinary phthalate metabolites, and lung function. Finally, age (18~45, 45~60, and ≥60 years old), sex (man, woman), body mass index (BMI, <18.5, 18.5~25, 25~30, and ≥30 kg/m^2^), race (Hispanic: Mexican American and other Hispanic; non-Hispanic: non-Hispanic White, non-Hispanic Black, and other race), level of education (below high school, high school, above high school), smoking (current, former, never), history of diabetes (yes, no), history of hypertension (yes, no), lung function, concentration of urinary phthalate metabolites (see the following definition), and count and percentage of blood cells were included.

### 2.3. Exposure of Phthalate

Urinary phthalate metabolites were used to evaluate the exposure of phthalate and its alternatives in humans. We included 12 kinds of urinary phthalate metabolites (mono carboxyisononyl phthalate, mono carboxyisoctyl phthalate, mono-2-ethyl-5-carboxypentyl phthalate, mono-n-butyl phthalate, mono-(3-carboxypropyl) phthalate, mono-ethyl phthalate, mono-(2-ethyl-5-hydroxyhexyl) phthalate, mono-(2-ethyl)-hexyl phthalate, mono-isobutyl phthalate, mono-isononyl phthalate, mono-(2-ethyl-5-oxohexyl) phthalate, and mono-benzyl phthalate), which were tested in all the NHANES cycles from 2007 to 2012. The methodology of detections for urinary phthalate metabolites was described on the NHANES official website (wwwn.cdc.gov/Nchs/Nhanes/2011-2012/PHTHTE_G.htm) (accessed on 1 February 2022). The value below the lower limit of detection was divided by square root of 2. Urinary MBP was used to evaluate the level of DBP exposure. In this study, the value of diethylhexyl phthalates was calculated as the sum of the concentrations of mono-(2-ethyl-5-carboxypentyl) phthalate, mono(2-ethyl-5-hydroxyhexyl) phthalate, and mono(2-ethyl-5-oxohexyl) phthalate. We used urinary creatinine-corrected urinary phthalate metabolites levels in the statistical models to reduce the differences of urinary phthalate metabolites caused by different urinary flow. Creatinine-adjusted urinary phthalate metabolites were also ln-transformed in the analysis. Compared with the group with normal lung function, MBP was the only significantly increased urinary phthalate metabolites in the group with declined lung function, so we used MBP as the main research object in the whole study.

### 2.4. Lung Function and Fractional Exhaled Nitrous Oxide

Spirometry was conducted for the included participants in this survey during 2007–2012; meanwhile, individual fractional exhaled nitrous oxide (FeNO) was also measured. The spirometers used in the study were Ohio 822/827 dry-rolling seal volume spirometers. The procedures of spirometry were based on the current standards for pulmonary function, equipment, testing, and interpretation set by the American Thoracic Society. The participants’ spirometry results were evaluated by a computerized algorithm. Examinees with an FEV1/FVC% less than the lower limit of normal determined for his or her age, sex, weight, height, and race/ethnicity or those who had a FEV1/FVC% less than 70% were considered as people with lung function impairment. Lower limit of normal for the FEV1/FVC was calculated based on the sample persons’ age, sex, height, and race/ethnicity [15]. The value of FeNO was calculated as the mean of two reproducible FeNO measurements in parts per billion (ppb). The FeNO data were used to estimate the airway inflammation, and the potential airway inflammation was defined as FeNO ≥ 25 ppb. The methodology about spirometry was described on the NHANES official website (wwwn.cdc.gov/Nchs/Nhanes/2011-2012/SPXRAW_G.htm) (accessed on 1 February 2022).

### 2.5. Daily Dietary Intake of Riboflavin

The 24 h detailed dietary intake information was collected in the dietary interview. United States Department of Agriculture’s Food and Nutrient Database for Dietary Studies (FNDDS) was used to calculate the nutrient intakes (http://www.ars.usda.gov/ba/bhnrc/fsrg) (accessed on 1 February 2022). The FNDDS includes comprehensive information that can be used to code individual foods and portion sizes reported by participants and also includes nutrient values for calculating nutrient intakes. Foods/beverages or portions that could not be matched to items in the database were resolved by scientists. Finally, the daily dietary intake of riboflavin (DDIR) in milligram (mg) was calculated from the total food and beverages consumption. The median of DDIR was 1.83 mg in this study, and the recommended intake of daily intake of riboflavin was 1.4~1.8 mg, and therefore, we defined high DDIR as DDIR ≥ 1.8 mg.

### 2.6. Statistical Analysis

Continuous variables were presented as means ± standard deviation (SD). Categorical variables were analyzed by the χ2 test or the Fisher’s exact test, as appropriate. Logistic regression was applied to estimate odds ratio (ORs) and 95% confidence intervals (CIs) for the association between risk of lung function impairment and urinary MBP. We divided the included participants into quartiles of ln(MBP), with the lowest quartile being the reference group. The linear trends in risk estimates across quartiles of ln(MBP/UCr) were calculated by one degree-of-freedom trend test [16]. We adjusted for several confounders in our logistic models. Specifically, model 1 was unadjusted, model 2 was adjusted for characteristics (sex, age, race, education); model 3 was adjusted for characteristics and underlying diseases (sex, age, race, education, sex, BMI, history of diabetes, smoke, history of hypertension, and respiratory diseases); and model 4 was adjusted for variables with significant *p*-values of linear trends (sex, age, race, education, smoke, history of hypertension, and respiratory diseases). Prespecified subgroup analyses were conducted to assess whether the observed association of risk of lung function impairment and urinary MBP was modified by sex, age, race, education, sex, BMI, history of diabetes, smoke, history of hypertension, respiratory diseases, and DDIR status. The ORs were adjusted for sex, age, race, education, smoke, history of hypertension, and respiratory diseases in subgroup analysis. A *P*_interaction_ was calculated by a likelihood ratio test, which compared the models with and without interaction terms. Restricted cubic spline regression with 3 knots at the 10th, 50th, and 90th percentiles was used to explore the potential dose–response relationship between ln(MBP/UCr) and lung function impairment. The reference level was set at 6.31. A *P*_nonlinearity_ was obtained by testing the null hypothesis that regression coefficients of the second splines are equal to zero. *p* < 0.05 was considered to indicate statistical significance. All statistical analyses were performed using SPSS 25.0 software and R 4.10 software.

## 3. Results

### 3.1. Characteristics of the Included Population

**Figure 1:**
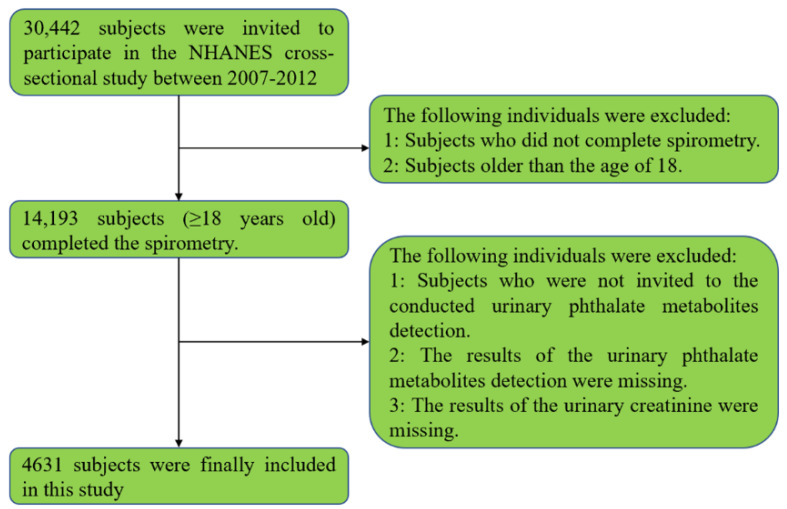
The study flow chart of identifying eligible subjects.

In general, 30,422 subjects were invited to participate in the NHANES cross-sectional study between 2005 and 2018. Participants without the results of urinary phthalate metabolites and spirometry were excluded. 4631 participants aged ≥18 years old were finally included in this study. The detailed information of screening process was shown in Figure 1. Participants aged 18–45 years old accounted for the highest proportion of all the study population (50.62%). The mean ± SD of DDIR in all participants was 2.10 ± 1.35 mg. There were 3889 cases (1943 men and 1944 women) in the normal group and 742 cases (432 men and 310 women) in the lung function impairment group (Table 1, Tables S1 and S2). There were significant differences of age, BMI, race, education, alcoholic drinks, current smoking, history of diabetes, and history of hypertension between the normal and lung function impairment groups. Among 12 kinds of urinary phthalate metabolites, only MBP was significantly higher in the lung function impairment group compared with the normal group (Table S1).

### 3.2. Subjects with Different Level of Urinary MBP

We divided study population into 4 groups by the quartiles of ln(MBP(ng/L)/UCr(g/dL)). The clinical characteristics, lung function, and the blood cells of the four groups are shown in Table 2. Participants in the highest vs. the lowest quartiles of MBP were younger; more likely to be non-Hispanic White; more likely to have a higher BMI, higher eosinophils, and neutrophils number and percent; and more likely to have a higher risk of respiratory diseases and lung function impairment (all *P*_trend_ < 0.05). (Table 2 and Table S3). However, there was no linear trend of FeNO among four groups (*P*_trend_ = 0.092).

### 3.3. Effects of DBP Exposure on Lung Function Impairment and Blood Cells

**Figure 2:**
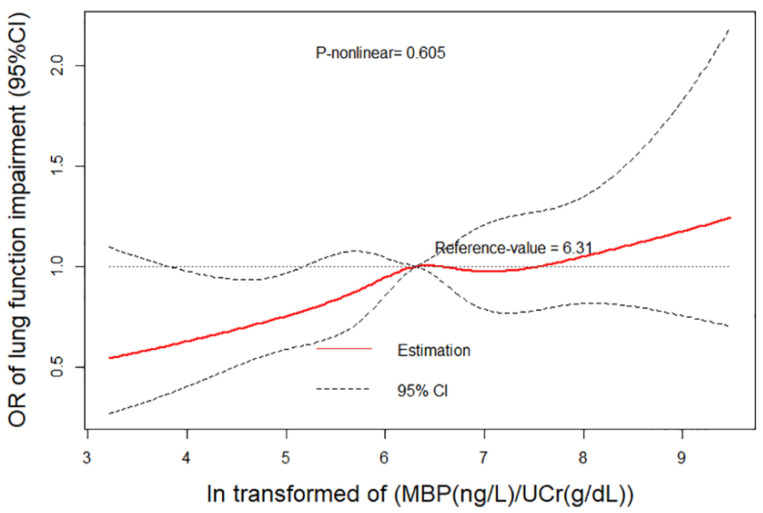
Non-linear dose–response analysis on urinary MBP concentration and lung function impairment. Restricted cubic spline regression with three knots at the 10th, 50th, and 90th percentiles was used to explore the potential dose–response relationship between MBP and lung function impairment. The reference level was set at 6.31. A *P*_non-linearity_ was obtained by testing the null hypothesis that regression coefficient of the second spline was equal to zero. CI, confidence interval; MBP, mono-benzyl phthalate; OR, odds ratio.

We have performed a dose–response analysis of the relationship between ln(MBP/UCr) and lung function impairment. It was observed that the higher concentration of MBP in urine was consistent with higher risk of all of lung function impairment (Figure 2). However, the *p*-value for the nonlinear dose–response manner was insignificant (*P*_nonlinear_ = 0.605).

The associations of DBP exposure and lung function impairment among the four groups are shown in Table 3. The results were consistent whether confounding factors were adjusted or not, and participants in the highest vs. the lowest quartiles of ln(MBP/UCr) were found to be at increased risks of lung function impairment. After the full adjustment for confounders, the result of lung function impairment was significantly higher in the highest MBP group compared with the lowest MBP group (OR _quartiles 4 vs. 1_ 1.35, 95% CI, 1.05–1.73; *P*_trend_ = 0.017). However, the results showed that there was no significant difference of FeNO between the highest MBP group vs. the lowest MBP group (OR _quartiles 4 vs. 1_ 1.17, 95% CI, 0.92–1.50; *P*_trend_ = 0.251).

The associations of MBP and four kinds of blood cells among the four groups are shown in Table 3 and Table S4. The results showed that there was significant difference of monocyte and neutrophil between higher MBP groups and quartiles 1 group. After the full adjustment for confounders, the ORs of higher monocytes and neutrophils were (OR _quartiles 4 vs. 1_ 1.37, 95% CI, 1.02–1.84; *P*_trend_ = 0.048) and (OR _quartiles 4 vs. 1_ 1.66, 95% CI, 1.21–2.27; *P*_trend_ = 0.008), respectively, in the highest vs. the lowest quartiles of ln(MBP/UCr).

### 3.4. Subgroup Analyses

The subgroup analyses on the associations of concentration of urinary MBP and lung function impairment (highest vs. the lowest quartiles of ln(MBP/UCr)) are shown in Table 4. A significant interaction between urinary MBP and DDIR (*P*_interaction_ = 0.029) and history of hypertension (*P*_interaction_ = 0.031) was detected for lung function impairment. Specifically, the highest fourth of MBP was found to be associated with higher risks of lung function impairment (OR _quartiles 4 vs. 1_, 1.64; 95% CI, 1.17–2.31) in people without hypertension; lower risk of lung function impairment (OR _quartiles 4 vs. 1_, 1.06; 95% CI, 0.74–1.54) was found in participants with higher DDIR. However, no significant interaction was found for remaining predefined factors.

### 3.5. Relationship between DDIR and Lung Function Impairment Related with DBP Exposure

Two hundred and two participants were excluded due to the miss data of DDIR, and 4429 participants were included in the comprehensive subgroup analyses of the of DDIR (Table 5). These participants were divided into low/normal DDIR group (*n* = 2178) and higher DDIR group (*n* = 2251). The effects of MBP on lung function impairment, peripheral monocytes, and neutrophils were evaluated in the two groups, respectively. After the full adjustment for confounders, the result of lung function impairment was significantly higher in the highest MBP group compared with lowest MBP group (OR _quartiles 4 vs. 1_ 1.85, 95% CI, 1.27–2.71; *P*_trend_ = 0.002) in the participants with low/normal DDIR. However, for participants with high DDIR, the highest vs. the lowest quartiles of MBP were not found to be at increased risks of lung function impairment (OR _quartiles 4 vs. 1_ 1.07, 95% CI, 0.75–1.53; *P*_trend_ = 0.616). The risk of high monocytes was not significant higher in the highest vs. the lowest quartiles of MBP in participants with low/normal DDIR but was significantly higher in the highest vs. the lowest quartiles of MBP (OR _quartiles 4 vs. 1_ 1.37, 95% CI, 1.00–1.87; *P*_trend_ = 0.049) in participants with higher DDIR. The risk of high neutrophils was significantly higher in the highest vs. the lowest quartiles of MBP (OR _quartiles 4 vs. 1_ 1.45, 95% CI, 1.06–1.97; *P*_trend_ = 0.018) in participants with low/normal DDIR but insignificantly higher (OR _quartiles 4 vs. 1_ 1.30, 95% CI, 0.97–1.75; *P*_trend_ = 0.119) in participants with high DDIR.

## 4. Discussion

In this national cross-sectional study, we revealed significant associations of concentration of urinary MBP with the risk of lung function impairment. Moreover, higher monocytes and neutrophils may contribute to lung function impairment associated with DBP exposure. Subgroup analyses further showed that these harmful associations between urinary MBP and lung function impairment were insignificant in people with high DDIR. High DDIR might reduce the neutrophil recruitment to alleviate the lung function impairment in the presence of DBP exposure.

Inhalation of DBP contributes to a large proportion of DBP intake due to the high concentration of DBP in indoor air. MBP is one of the main DBP metabolites [17], and MBP was the only significantly higher urinary phthalate metabolites in participants with lung function impairment compared with participants without lung function impairment in our study. The similar results of the harmful association between MBP and lung function impairment was shown in one previous study [18]. However, it was suggested that DBP exposure increased the early allergic response in that study, but the results of our studies showed that the FeNO and eosinophils were not significantly higher in the group with higher MBP than lower MBP. The inconsistency might be related with the small sample size in the previous study, which only included 16 participants.

In our study, we found that monocytes and neutrophils were higher in the high urinary MBP groups, and the trend of the changes in monocytes and neutrophils were also significant. Therefore, urinary MBP level was harmfully associated with changes in lung function and might be mediated by monocytes and neutrophils. One previous study reported the interaction between DBP and macrophages was mediated through PPARγ, which was highly expressed in macrophages [19,20]. As we know, the blood monocytes migrated into lung tissue and gave rise to interstitial and alveolar macrophages. Therefore, the changes of the peripheral monocytes might be related with the change of macrophages in lung effected by DBP [21]. It was reported that number of neutrophils in lavage fluid of mice in phthalates group was higher compared with the groups without phthalates [22]. Migration of blood neutrophils into the lung tissue is an important process of immune reaction, and neutrophils in lung tissue can also migrate into blood in patients with COPD [23]. It was reported that the phthalate treatment leaded to elevation of oxidant stress in neutrophils [24]. However, one previous study showed that riboflavin exerted both the antioxidant and anti-inflammatory effects against acetic acid-induced colonic inflammation by suppressing neutrophil accumulation, inhibiting reactive oxidant generation [25]. The decreasing level of TGFβ and TNF-α and the increasing level of glutathione in neutrophil may be the potential mechanism of antioxidant and anti-inflammatory effects of riboflavin [25,26]. Therefore, we speculated that high daily intake of riboflavin improves the lung function by suppressing neutrophil accumulation and inhibiting reactive oxidant generation induced by DBP exposure (Figure S1).

Interestingly, our subgroup analysis suggested that the lung function of participants with higher DDIR were less likely to be affected by DBP than patients with lower/normal DDIR. The relationship between riboflavin intake and lung function impairment associated with phthalates exposure is still unknown. Some previous studies suggested that riboflavin can suppress neutrophil accumulation, inhibit reactive oxidant generation, and improve oxidative DNA damage in a murine model of AA-induced colonic inflammation, sepsis and ischemia/reperfusion injury [25,27]. A similar result was also observed in our study. Suppressing neutrophil accumulation might be the main reason for the association of DDIR and lung function in the presence of DBP exposure.

There were several strengths in this study. Firstly, to our knowledge, this is the first population-based study to explore the relationship between DDIR and lung function impairment in the presence of DBP exposure. Secondly, we comprehensively analyzed the relationship between urinary MBP and lung function impairment and found the lung function impairment is associated with high levels of urinary MBP. Thirdly, this is the first study to reveal that the immuno-modulatory effects of riboflavin and changes of neutrophils might be the potential mechanism.

Limitations of this study should also be noted. Firstly, most measurements and questionnaires were completed at the same time, so we can only obtain clues rather than determine the direct causality in a large sample of population. Secondly, it is necessary to detect urinary MBP at different time points because the level of MBP at one time point cannot represent the long-term level of DBP exposure. Furthermore, we also need to follow up regarding the changes of lung function. Similarly, the 24 h dietary questionnaire made it difficult to accurately discern the persisting nutrition intake. Therefore, it is necessary to complete a daily diet questionnaire during a long period. Thirdly, we also need to test the serum riboflavin concentration to explore the true riboflavin exposure because the riboflavin will be excreted through urine when it is beyond a certain level. Finally, the actual relationship between the number of monocytes and neutrophils in blood and in alveolar lavage fluid should be explored, and further study can focus on the neutrophil accumulation in lung tissue in an experimental murine model of lung function impairment associated with DBP exposure.

In summary, our study suggests that higher concentration of urinary MBP was associated with higher risk of lung function impairment, and the harmful association might be mediated by monocytes and neutrophils. A positive association between high DDIR and lung function in the presence of DBP exposure was observed in this study, and the inhibiting of the neutrophil recruitment might be the potential mechanism.

## Figures and Tables

**Table 1:** The characteristics of study population and the difference between groups with and without lung function impairment.

	Total	Lung Function Impairment		*p*
		No	Yes	
**Sample size**	4631	3889	742	
**Sex (men, %)**	2375 (51.28%)	1943 (49.96%)	432 (58.22%)	<0.001
**Age (years old)**				<0.001
18–45	2344 (50.62%)	2100 (54.00%)	244 (32.88%)	
45–60	1135 (24.51%)	957 (24.61%)	178 (23.99%)	
≥60	1152 (24.88%)	832 (21.39%)	320 (43.13%)	
**BMI (kg/m^2^)**				<0.001
<18.5	83 (1.79%)	69 (1.78%)	14 (1.90%)	
18.5–25	1341 (28.96%)	1081 (27.96%)	260 (35.28%)	
25–30	1534 (33.12%)	1295 (33.50%)	239 (32.43%)	
≥30	1645 (35.52%)	1421 (36.76%)	224 (30.39%)	
**Race**				<0.001
Mexican American	734 (15.85%)	667 (17.15%)	67 (9.03%)	
Other Hispanic	483 (10.43%)	424 (10.90%)	59 (7.95%)	
Non-Hispanic White	1955 (42.22%)	1535 (39.47%)	420 (56.60%)	
Non-Hispanic Black	1031 (22.26%)	873 (22.45%)	158 (21.29%)	
Other Race	428 (9.24%)	390 (10.03%)	38 (5.12%)	
**Education**				0.014
Below high school	1187 (25.65%)	985 (25.35%)	202 (27.26%)	
High school	1078 (23.30%)	882 (22.70%)	196 (26.45%)	
Above high school	2362 (51.05%)	2019 (51.96%)	343 (46.29%)	
**Smoking status (yes, %)**				<0.001
Never	2383 (54.79%)	2140 (58.86%)	243 (34.08%)	
Former	977 (22.46%)	758 (20.85%)	219 (30.72%)	
Current	989 (22.74%)	738 (20.30%)	251 (35.20%)	
**Respiratory disease (yes, %)**	565 (12.20%)	396 (10.18%)	169 (22.78%)	<0.001
**History of diabetes (yes, %)**	466 (10.07%)	373 (9.60%)	93 (12.53%)	0.015
**History of hypertension (yes, %)**	1376 (29.74%)	1109 (28.54%)	267 (36.03%)	<0.001
**Mono-benzyl phthalate**	6.32 ± 0.98	6.30 ± 0.98	6.40 ± 0.96	0.007
**Lung function impairment**	742 (16.02%)	0 (0%)	742 (100%)	
**FeNO**	16.91 ± 15.05	16.67 ± 14.31	18.18 ± 18.39	0.039
**Blood cells**				
Monocyte number (cells/uL)	529.34 ± 183.65	524.46 ± 180.27	554.93 ± 198.65	<0.001
Neutrophils number (cells/uL)	4259.81 ± 2071.89	4227.04 ± 2127.74	4431.69 ± 1741.19	0.006
**Daily dietary intake of riboflavin (mg)**	2.10 ± 1.35	2.09 ± 1.33	2.19 ± 1.44	0.077

Lung function impairment: best test FEV1/FVC ratio below the lower limit of normal and/or less than 70%. BMI, body mass index; FeNO, fractional exhaled nitrous oxide.

**Table 2:** The clinical characteristics of study population according to the concentration of urinary MBP.

Characteristics	Quartile of ln(MBP/UCr), Range (Median)				*P*_trend_
	2.15~5.69 (5.23)	5.69~6.31 (6.03)	6.31~6.93 (6.59)	6.93~10.20 (7.40)	
**Sample size**	1164	1155	1151	1161	
**Sex (men, %)**	689 (59.19%)	615 (53.25%)	572 (49.70%)	499 (42.98%)	<0.001
**Age (years old)**					<0.001
18–45	564 (48.45%)	526 (45.54%)	589 (51.17%)	665 (57.28%)	
45–60	272 (23.37%)	316 (27.36%)	284 (24.67%)	263 (22.65%)	
≥60	328 (28.18%)	313 (27.10%)	278 (24.15%)	233 (20.07%)	
**BMI (kg/m^2^)**					0.715
<18.5	11 (0.90%)	21 (1.87%)	18 (1.72%)	33 (2.99%)	
18.5–25	339 (27.79%)	310 (27.60%)	351 (33.59%)	341 (30.89%)	
25–30	435 (35.66%)	396 (35.26%)	338 (32.34%)	365 (33.06%)	
≥30	373 (30.57%)	426 (37.93%)	434 (41.53%)	412 (37.32%)	
**Race**					<0.001
Mexican American	198 (17.01%)	158 (13.68%)	192 (16.68%)	186 (16.02%)	
Other Hispanic	126 (10.82%)	131 (11.34%)	109 (9.47%)	117 (10.08%)	
Non-Hispanic White	421 (36.17%)	466 (40.35%)	508 (44.14%)	560 (48.23%)	
Non-Hispanic Black	271 (23.28%)	282 (24.42%)	255 (22.15%)	223 (19.21%)	
Other Race	148 (12.71%)	118 (10.22%)	87 (7.56%)	75 (6.46%)	
**Education**					<0.001
Below high school	258 (22.18%)	271 (23.46%)	319 (27.72%)	339 (29.27%)	
High school	236 (20.29%)	255 (22.08%)	301 (26.15%)	286 (24.70%)	
Above high school	669 (57.52%)	629 (54.46%)	531 (46.13%)	533 (46.03%)	
**Smoking status (yes, %)**					<0.001
Never	640 (57.92%)	608 (55.37%)	583 (54.38%)	552 (51.40%)	
Former	276 (24.98%)	261 (23.77%)	245 (22.85%)	195 (18.16%)	
Current	189 (17.10%)	229 (20.86%)	244 (22.76%)	327 (30.45%)	
**Respiratory disease (yes, %)**	128 (11.00%)	131 (11.34%)	142 (12.34%)	164 (14.13%)	0.016
**History of diabetes (yes, %)**	114 (9.80%)	127 (11.01%)	120 (10.43%)	105 (9.04%)	0.470
**History of hypertension (yes, %)**	362 (31.10%)	364 (31.57%)	334 (29.07%)	316 (27.22%)	0.018
**Lung function impairment**	155 (13.32%)	187 (16.19%)	200 (17.38%)	200 (17.23%)	0.007
**FeNO**	197 (17.78%)	183 (16.73%)	170 (15.47%)	168 (15.40%)	0.092
**Monocyte number (cells/uL)**	510.29 ± 172.93	519.58 ± 178.28	540.40 ± 186.68	547.01 ± 193.75	<0.001
**Neutrophils number (cells/uL)**	4028.43 ± 1513.40	4172.29 ± 1756.88	4298.82 ± 1759.86	4537.27 ± 2918.24	<0.001

ln(MBP/UCr), ln(mono-benzyl phthalate(ng/L)/urinary creatinine(g/dL)); MBP, mono-benzyl phthalate; lung function impairment, best test FEV1/FVC ratio below lower limit of normal and/or less than 70%; BMI, body mass index; FeNO, fractional exhaled nitrous oxide.

**Table 3:** Association between urinary MBP and the lung function impairment.

Outcomes	Model	Quartiles of ln(MBP/UCr), Range (Median)				*P*_trend_
		2.15~5.69 (5.23)	5.69~6.31 (6.03)	6.31~6.93 (6.59)	6.93~10.20 (7.40)	
Lung function impairment	Model 1	1.00 (Reference)	1.26 (1.00, 1.58)	1.37 (1.09, 1.72)	1.35 (1.08, 1.70)	0.007
	Model 2	1.00 (Reference)	1.23 (0.97, 1.56)	1.36 (1.08, 1.73)	1.44 (1.14, 1.83)	0.002
	Model 3	1.00 (Reference)	1.25 (0.98, 1.61)	1.35 (1.05, 1.73)	1.36 (1.06, 1.75)	0.015
	Model 4	1.00 (Reference)	1.24 (0.97, 1.59)	1.34 (1.05, 1.72)	1.35 (1.05, 1.73)	0.017
High monocyte	Model 1	1.00 (Reference)	1.20 (0.97, 1.48)	1.47 (1.20, 1.81)	1.43 (1.16, 1.76)	<0.001
	Model 2	1.00 (Reference)	1.20 (0.96, 1.49)	1.42 (1.15, 1.76)	1.40 (1.13, 1.74)	0.001
	Model 3	1.00 (Reference)	1.25 (0.99, 1.56)	1.37 (1.10, 1.72)	1.29 (1.02, 1.62)	0.023
	Model 4	1.00 (Reference)	1.27 (1.02, 1.60)	1.41 (1.13, 1.77)	1.32 (1.05, 1.66)	0.011
High neutrophil	Model 1	1.00 (Reference)	1.16 (0.95, 1.41)	1.32 (1.08, 1.60)	1.66 (1.37, 2.01)	<0.001
	Model 2	1.00 (Reference)	1.14 (0.93, 1.39)	1.21 (0.99, 1.48)	1.46 (1.20, 1.78)	<0.001
	Model 3	1.00 (Reference)	1.13 (0.92, 1.40)	1.16 (0.94, 1.44)	1.29 (1.04, 1.59)	0.022
	Model 4	1.00 (Reference)	1.16 (0.94, 1.43)	1.20 (0.98, 1.49)	1.33 (1.08, 1.64)	0.008

ln(MBP/UCr), ln(mono-benzyl phthalate(ng/L)/urinary creatinine(g/dL)); MBP, mono-benzyl phthalate; lung function impairment, best test FEV1/FVC ratio below lower limit of normal and/or less than 70%; high monocyte, the number of monocyte ≥1000 cells/ul or the monocyte percent ≥10%; high neutrophil, the number of neutrophil ≥7000 cells/ul or the monocyte percent ≥70%; Model 1, unadjusted model; Model 2, adjusted for gender (man or woman), age (years old), race (Mexican American, other Hispanic, Non-Hispanic White, Non-Hispanic Black, and other race), and education (below high school, high school, above high school); Model 3, adjusted for gender (man or woman), age (years old), race (Mexican American, other Hispanic, Non-Hispanic White, Non-Hispanic Black, and other race), education (below high school, high school, above high school), BMI (kg/m^2^), history of diabetes (yes or no), smoker status (never, former, current), and hypertension (yes or no); Model 4, adjusted for gender (man or woman), age (years old), race (Mexican American, other Hispanic, Non-Hispanic White, Non-Hispanic Black, and other race), education (below high school, high school, above high school), history of diabetes (yes or no), and hypertension (yes or no).

**Table 4:** Subgroup analyses on the association between urinary MBP and lung function impairment.

Subgroup Variable	OR _quartile 4 vs. 1_ (95% CI)	*p*	*P*_interaction_
**Sex**			
woman	1.33 (0.90, 2.00)	0.160	0.752
man	1.35 (0.94, 1.93)	0.102	
**Age (years old)**			
18–45	1.20 (0.76, 1.90)	0.435	
45–60	1.62 (0.94, 2.82)	0.082	0.451
≥60	1.36 (0.88, 2.09)	0.166	0.975
**Race**			
Mexican American	1.07 (0.51, 2.26)	0.855	
Other Hispanic	4.25 (1.40, 14.53)	0.142	0.309
Non-Hispanic White	1.57 (1.08, 2.29)	0.020	0.406
Non-Hispanic Black	1.51 (0.85, 2.70)	0.161	0.950
Other Race	0.35 (0.06, 1.40)	0.172	0.164
**Education**			
Below high school	1.45 (0.84, 2.54)	0.181	
High school	1.45 (0.84, 2.55)	0.183	0.834
Above high school	1.40 (0.97, 2.04)	0.075	0.625
**History of diabetes**			
No	1.46 (1.10, 1.94)	0.009	
Yes	0.84 (0.38, 1.85)	0.670	0.126
**Respiratory disease**			
No	1.47 (1.09, 1.98)	0.012	
Yes	1.05 (0.58, 1.90)	0.933	0.409
**Smoke status**			
Never	1.70 (1.10, 2.63)	0.017	
Former	1.46 (0.87, 2.44)	0.154	0.474
Current	1.08 (0.68, 1.73)	0.748	0.232
**History of hypertension**			
No	1.64 (1.17, 2.31)	0.006	
Yes	1.05 (0.68, 1.62)	0.999	0.031
**Daily dietary intake of riboflavin (mg)**			
Low/normal (≤1.8)	1.85 (1.25, 2.75)	0.004	
Higher (>1.8)	1.07 (0.74, 1.54)	0.717	0.026

ln(MBP/UCr), ln(mono-benzyl phthalate(ng/L)/urinary creatinine(g/dL)); MBP, mono-benzyl phthalate; lung function impairment, best test FEV1/FVC ratio below lower limit of normal and/or less than 70%; BMI, body mass index; ORs were adjusted for gender (man or woman), age (years old), race (Hispanic: Mexican American and other Hispanic, non-Hispanic: non-Hispanic White, non-Hispanic Black, and other race), education (below high school, high school, above high school), history of diabetes (yes or no), and hypertension (yes or no).

**Table 5:** Association between urinary MBP and the lung function impairment, neutrophils, and monocytes in different level of daily dietary intake of riboflavin.

	Outcomes	Model	Quartile of ln(MBP/UCr), Range (Median)				*P*_trend_
			2.15~5.69 (5.23)	5.69~6.31 (6.03)	6.31~6.93 (6.59)	6.93~10.20 (7.40)	
Low/normal DDIR (*n* = 2178)	Lung function impairment	Model 1	1.00 (Reference)	1.59 (1.12, 2.26)	1.63 (1.15, 2.31)	1.80 (1.28, 2.54)	0.002
		Model 2	1.00 (Reference)	1.43 (1.00, 2.05)	1.54 (1.08, 2.21)	1.94 (1.36, 2.79)	<0.001
		Model 3	1.00 (Reference)	1.41 (0.97, 2.06)	1.40 (0.96, 2.05)	1.85 (1.27, 2.70)	0.003
		Model 4	1.00 (Reference)	1.41 (0.97, 2.06)	1.44 (0.99, 2.11)	1.85 (1.27, 2.71)	0.002
	High neutrophils	Model 1	1.00 (Reference)	1.22 (0.91, 1.65)	1.47 (1.10, 1.97)	1.73 (1.30, 2.30)	<0.001
		Model 2	1.00 (Reference)	1.27 (0.94, 1.72)	1.39 (1.04, 1.87)	1.56 (1.17, 2.09)	0.003
		Model 3	1.00 (Reference)	1.28 (0.93, 1.76)	1.33 (0.97, 1.82)	1.42 (1.04, 1.95)	0.032
		Model 4	1.00 (Reference)	1.26 (0.92, 1.73)	1.37 (1.00, 1.87)	1.45 (1.06, 1.97)	0.018
	High monocytes	Model 1	1.00 (Reference)	1.11 (0.80, 1.54)	1.37 (1.00, 1.88)	1.42 (1.04, 1.94)	0.013
		Model 2	1.00 (Reference)	1.12 (0.80, 1.56)	1.30 (0.94, 1.80)	1.42 (1.03, 1.97)	0.021
		Model 3	1.00 (Reference)	1.13 (0.80, 1.61)	1.23 (0.87, 1.74)	1.22 (0.87, 1.73)	0.225
		Model 4	1.00 (Reference)	1.14 (0.81, 1.62)	1.26 (0.89, 1.77)	1.24 (0.88, 1.75)	0.193
High DDIR (*n* = 2251)	Lung function impairment	Model 1	1.00 (Reference)	1.12 (0.80, 1.55)	1.18 (0.86, 1.63)	1.09 (0.78, 1.51)	0.570
		Model 2	1.00 (Reference)	1.14 (0.81, 1.61)	1.26 (0.90, 1.77)	1.15 (0.82, 1.63)	0.350
		Model 3	1.00 (Reference)	1.20 (0.84, 1.72)	1.32 (0.93, 1.89)	1.10 (0.76, 1.58)	0.531
		Model 4	1.00 (Reference)	1.17 (0.82, 1.66)	1.29 (0.91, 1.83)	1.07 (0.75, 1.53)	0.616
	High neutrophils	Model 1	1.00 (Reference)	1.15 (0.87, 1.52)	1.22 (0.92, 1.61)	1.61 (1.23, 2.11)	0.001
		Model 2	1.00 (Reference)	1.07 (0.80, 1.42)	1.08 (0.82, 1.44)	1.39 (1.05, 1.83)	0.024
		Model 3	1.00 (Reference)	1.10 (0.81, 1.49)	1.06 (0.78, 1.44)	1.25 (0.92, 1.68)	0.195
		Model 4	1.00 (Reference)	1.16 (0.86, 1.56)	1.11 (0.82, 1.49)	1.30 (0.97, 1.75)	0.119
	High monocytes	Model 1	1.00 (Reference)	1.24 (0.93, 1.66)	1.50 (1.13, 1.99)	1.38 (1.04, 1.85)	0.012
		Model 2	1.00 (Reference)	1.21 (0.90, 1.63)	1.44 (1.08, 1.93)	1.34 (0.99, 1.80)	0.031
		Model 3	1.00 (Reference)	1.28 (0.94, 1.75)	1.40 (1.03, 1.92)	1.34 (0.98, 1.83)	0.065
		Model 4	1.00 (Reference)	1.34 (0.99, 1.83)	1.45 (1.07, 1.97)	1.37 (1.00, 1.87)	0.049

ln(MBP/UCr), ln(mono-benzyl phthalate(ng/L)/urinary creatinine(g/dL)); MBP, mono-benzyl phthalate; lung function impairment, best test FEV1/FVC ratio below lower limit of normal and/or less than 70%; DDIR, daily dietary intake of riboflavin; high monocyte, the number of monocyte ≥1000 cells/ul or the monocyte percent ≥10%; high neutrophil, the number of neutrophil ≥7000 cells/ul or the monocyte percent ≥70%; Model 1, Unadjusted model; Model 2, Adjusted for gender (man or woman), age (years old), race (Mexican American, other Hispanic, Non-Hispanic White, Non-Hispanic Black, and other race), and education (below high school, high school, above high school); Model 3, adjusted for gender (man or woman), age (years old), race (Mexican American, other Hispanic, Non-Hispanic White, Non-Hispanic Black, and other race), education (below high school, high school, above high school), BMI (kg/m^2^), history of diabetes (yes or no), smoker status (never, former, current), and hypertension (yes or no); Model 4, adjusted for gender (man or woman), age (years old), race (Mexican American, other Hispanic, Non-Hispanic White, Non-Hispanic Black, and other race), education (below high school, high school, above high school), history of diabetes (yes or no), and hypertension (yes or no).

## Data Availability

All of original data used in this study are freely available to the public. This data can be found here: https://www.cdc.gov/nchs/nhanes/ (accessed on 1 February 2022). All code of R software of this study are available from the corresponding author upon reasonable request.

## Supplementary Materials

The following supporting information can be downloaded at: https://www.mdpi.com/article/10.3390/nu14112282/s1, Table S1: The urinary phthalate metabolites of study population and the difference between groups with and without lung function impairment; Table S2: The blood cells of study population and the difference between groups with and without lung function impairment; Table S3: The blood cells of study population according to the concentration of urinary MBP; Table S4: Association between FeNO, eosinophil and urinary MBP; Figure S1: The potential biochemical and cell biological mechanisms of the riboflavin on lung function. Riboflavin exerted both the antioxidant and anti-inflammatory effects against acetic acid-induced colonic inflammation by suppressing neutrophil accumulation, inhibiting reactive oxidant generation. The decreasing level of TGFβ and TNF-α and increasing levels of glutathione in neutrophil may be the potential antioxidant and anti-inflammatory effects of riboflavin.
