# Supplementary material for: The Association between Daily Dietary Intake of Riboflavin and Lung Function Impairment Related with Dibutyl Phthalate Exposure and the Possible Mechanism
Source: Nutrients. 2022 May 29;14(11):2282. doi: 10.3390/nu14112282 (PMC9182752; doi:10.3390/nu14112282)
Supplement: Supplementary file 1 [file nutrients-14-02282-s001.zip › nutrients-1687486-supplementary.pdf]

Table S1: The urinary phthalate metabolites of study population and the difference between groups with and without lung function impairment

	Total	Lung function impairment		P
		No	Yes	
Mono carboxyisononyl phthalate	5.51 ±0.95	5.51 ±0.96	5.49 ±0.90	0.488
Mono carboxyoctyl phthalate	7.01 ±1.28	7.02 ±1.27	6.94 ±1.29	0.113
Mono-2-ethyl-5-carboxypentyl phthalate	7.59 ±0.99	7.59 ±0.99	7.60 ±0.98	0.751
Mono-n-butyl phthalate	7.18 ±0.97	7.18 ±0.96	7.19 ±0.97	0.871
Mono-(3-carboxypropyl) phthalate	5.59 ±1.08	5.58 ±1.09	5.64 ±1.00	0.120
Mono-ethyl phthalate	8.95 ±1.43	8.94 ±1.43	8.99 ±1.38	0.333
Mono-(2-ethyl-5-hydroxyhexyl) phthalate	7.15 ±1.06	7.15 ±1.07	7.17 ±1.03	0.564

Mono-(2-ethyl)-hexyl phthalate	5.22 ±1.12	5.24 ±1.13	5.14 ±1.06	0.027
Mono-isobutyl phthalate	6.58 ±0.82	6.60 ±0.81	6.49 ±0.83	0.001
Mono-isononyl phthalate	4.68 ±1.15	4.69 ±1.15	4.66 ±1.12	0.531
Mono-(2-ethyl-5-oxohexyl) phthalate	6.64 ±1.01	6.64 ±1.01	6.66 ±0.99	0.599
Mono-benzyl phthalate	6.32 ±0.98	6.30 ±0.98	6.40 ±0.96	0.007
DEHP	8.38 ±0.97	8.38 ±0.98	8.39 ±0.95	0.733

Lung function impairment: decline in lung function: best test FEV1/FVC ratio below lower limit of normal and/or less than 70%. DEHP: di-2-ethylhexyl phthalate, DEHP=Mono-2-ethyl-5-carboxypentyl phthalate+Mono-(2-ethyl-5-hydroxyhexyl) phthalate, +Mono-(2-ethyl)-hexyl phthalate +Mono-(2-ethyl-5-oxohexyl) phthalate. UPMs: urinary phthalate metabolites. The ln transformed of [urinary phthalate metabolites (ng/L)/ urinary creatinine (g/dL)] were used in the analysis.

Table S2: The blood cells of study population and the difference between groups with and without lung function impairment

	Total	Lung function impairment		P
		No	Yes	
**Blood cells**				
Monocyte percent (%)	7.62 ±2.27	7.60 ±2.26	7.71 ±2.33	0.261
Neutrophils percent (%)	57.92 ±9.51	57.79 ±9.46	58.61 ±9.73	0.041
Eosinophils percent (%)	2.82 ±2.08	2.79 ±2.10	2.98 ±1.95	0.024
Basophils percent (%)	0.69 ±0.55	0.69 ±0.55	0.69 ±0.58	0.825
Monocyte number (cells/uL)	529.34 ±183.65	524.46 ±180.27	554.93 ±198.65	<0.001
Neutrophils number (cells/uL)	4259.81 ±2071.89	4227.04 ±2127.74	4431.69 ±1741.19	0.006
Eosinophils number (cells/uL)	200.14 ±200.36	197.53 ±208.85	213.8 ±147.40	0.012
Basophils number (cells/uL)	40.41 ±93.58	40.09 ±98.56	42.11 ±61.15	0.471

Lung function impairment: best test FEV1/FVC ratio below lower limit of normal and/or less than 70%.

Table S3: The blood cells of study population according to the concentration of urinary MBP

Blood cells	Quartile of ln(MBP/Ucr), range (median)				P_{trend}
	**2.15~5.69 (5.23)**	**5.69~6.31 (6.03)**	**6.31~6.93 (6.59)**	**6.93~10.20 (7.40)**	
**Monocyte percent (%)**	7.64 ±2.15	7.6 ±2.29	7.70 ±2.35	7.53 ±2.30	0.463
**Neutrophils percent (%)**	57.46 ±8.98	57.8 ±9.90	57.96 ±9.66	58.47 ±9.45	0.012
**Eosinophils percent (%)**	2.76 ±1.88	2.88 ±2.19	2.82 ±1.98	2.83 ±2.23	0.633
**Basophils percent (%)**	0.72 ±0.57	0.69 ±0.58	0.66 ±0.47	0.69 ±0.59	0.116
**Monocyte number**	510.29 ±172.93	519.58 ±178.28	540.4 ±186.68	547.01 ±193.75	<0.001

**(cells/uL)**					
**Neutrophils number (cells/uL)**	4028.43 ±1513.40	4172.29 ±1756.88	4298.82 ±1759.86	4537.27 ±2918.24	<0.001
**Eosinophils number (cells/uL)**	189.62 ±145.24	196.66 ±161.12	200.09 ±149.04	214.03 ±300.76	0.004
**Basophils number (cells/uL)**	39.89 ±60.69	39.17 ±63.05	37.94 ±65.9	44.59 ±151.16	0.301

ln(MBP/Ucr): ln[mono-benzyl phthalate(ng/L)/urinary creatinine(g/dL)]; MBP: mono-benzyl phthalate.

Table S4: Association between FeNO, eosinophil and urinary MBP

Outcomes	Model	Quartiles of ln(MBP/UCr), range (median)				P_{trend}
		2.15~5.69 (5.23)	5.69~6.31 (6.03)	6.31~6.93 (6.59)	6.93~10.20 (7.40)	
FeNO	Model1	1.00 (Reference)	0.93 (0.74, 1.16)	0.85 (0.68, 1.06)	0.84 (0.67, 1.05)	0.092
	Model2	1.00 (Reference)	0.99 (0.79, 1.24)	0.98 (0.78, 1.23)	1.05 (0.83, 1.32)	0.737
	Model3	1.00 (Reference)	1.08 (0.85, 1.36)	1.06 (0.83, 1.35)	1.17 (0.92, 1.50)	0.243
	Model4	1.00 (Reference)	1.07 (0.85, 1.36)	1.06 (0.83, 1.34)	1.17 (0.92, 1.50)	0.251
High eosinophil	Model1	1.00 (Reference)	1.08 (0.82, 1.41)	1.00 (0.76, 1.31)	1.18 (0.91, 1.54)	0.300

Model2	1.00 (Reference)	1.11 (0.85, 1.46)	1.07 (0.81, 1.4)	1.31 (1.00, 1.71)	0.078
Model3	1.00 (Reference)	1.08 (0.82, 1.43)	1.00 (0.75, 1.33)	1.21 (0.91, 1.61)	0.275
Model4	1.00 (Reference)	1.09 (0.83, 1.44)	1.02 (0.77, 1.36)	1.23 (0.93, 1.63)	0.222

ln(MBP/UCr): ln[mono-benzyl phthalate(ng/L)/urinary creatinine(g/dL)]; MBP: mono-benzyl phthalate; FeNO: fractional exhaled nitrous oxide; high eosinophil: the number of eosinophil >=500 cells/ul or the eosinophil percent >=5%;

Model1: Unadjusted model

Model2: Adjusted for gender (man or woman), age (years old), race(Mexican American, other Hispanic, Non-Hispanic White, Non-Hispanic Black, and Other Race), education (below high school, high school, above high school)

Model3: Adjusted for gender (man or woman), age (years old), race(Mexican American, other Hispanic, Non-Hispanic White, Non-Hispanic Black, and Other Race), education (below high school, high school, above high school), BMI (kg/m2), history of diabetes (yes or no), smoker status(never,former, current), and hypertension(yes or no).

Model4: Adjusted for gender (man or woman), age (years old), race(Mexican American, other Hispanic, Non-Hispanic White, Non-Hispanic Black, and Other Race), education (below high school, high school, above high school), history of diabetes (yes or no), and hypertension(yes or no).

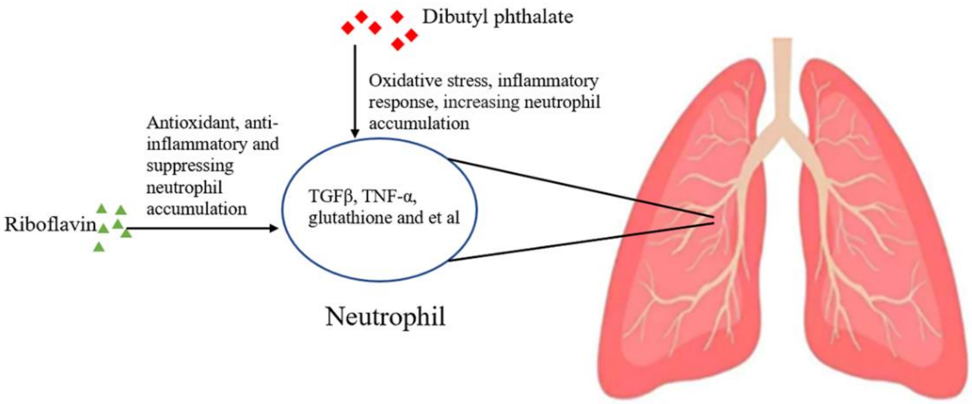

Figure S1: the potential biochemical and cell biological mechanisms of the riboflavin on lung function. Riboflavin exerted both the antioxidant and anti-inflammatory effects against acetic acid-induced colonic inflammation by suppressing neutrophil accumulation, inhibiting reactive oxidant generation. The decreasing level of TGFβ and TNF-α, and increasing levels of glutathione in neutrophil may be the potential antioxidant and anti-inflammatory effects of riboflavin.
